# Standard and Newly Defined Prognostic Factors Affecting Early Mortality After Hip Fractures

**DOI:** 10.7759/cureus.21464

**Published:** 2022-01-21

**Authors:** Necmettin Turgut, Abdullah Meriç Ünal

**Affiliations:** 1 Orthopedics and Traumatology, Isparta City Hospital, Isparta, TUR; 2 Orthopedics and Traumatology, Özel Meddem Hospital, Isparta, TUR

**Keywords:** early mortality, hip fracture, platelet/lymphocyte ratio, neutrophil/lymphocyte ratio, nonagenarian

## Abstract

Purpose: Early mortality rate in geriatric patients after hip fractures remains very high. Determining the prognostic factors is crucial for decreasing early mortality. This study aimed to evaluate the prognostic risk factors affecting early mortality after hip fracture in the elderly.

Methods: Medical records of 335 patients with age 70 years or older who sustained hip fractures which were treated by hemiarthroplasty or proximal femoral nailing between May 2017 and May 2019 were reviewed. Neutrophil/lymphocyte ratio (NLR) and platelet/lymphocyte ratio (PLR) were investigated for validity as the new prognostic markers. The other variables included age, gender, type of surgery, type of implant, type of anesthesia, American Society of Anesthesiologists (ASA) score, presence of comorbidities, delirium, length of hospital stay, time delay to surgery, number of erythrocyte transfusions, and laboratory data were assessed for 30-day, 90-day, and one-year mortality. Univariate analysis and logistic regression analysis were used to determine the associated mortality.

Results: Thirty-day mortality rate was 10.4% and was associated with being aged ≥90 years (p-value: 0.013, odds ratio {OR}: 0.13) and ASA score of 4 (p-value: 0.019, OR: 0.22). Ninety-day mortality rate was 21.5% and was associated with age (p-value: 0.002), being aged 80-89 years (p-value: 0.032, OR: 0.43), being aged ≥90 years (p-value: 0.001, OR: 0.13), general anesthesia (p-value: 0.016, OR: 0.41), preoperative high NLR level (p-value: 0.028, OR: 1.05), high blood urea nitrogen (BUN) level (p-value: 0.049, OR:1.02). One year mortality rate was 33.7% and independent significant prognostic risk factors were determined as being aged ≥90 years (p-value: 0.003, OR: 0.23), length of hospital stay (p-value: 0.003, OR: 1.04), and preoperative serum albumin level (p-value: 0.037, OR: 0.6). The other evaluated risk factors were not independently found to be associated with all early mortality time.

Conclusion: Patients at or over 90 years old were at risk for all early mortality time points. NLR which is a new and cheap biomarker can be used as a prognostic risk factor for 90-day mortality. The variable of PLR was not found valuable for early mortality.

## Introduction

Hip fractures in elderly patients are a significant public health problem and mainly affect the elderly. It is strongly associated with disability and mortality, and it is well-known that mortality is increased after hip fracture, especially in the first postoperative year [1].

Identifying prognostic factors for mortality in patients with hip fracture can lead to proper management, which is essential if mortality is to be reduced. Several studies have been conducted to investigate mortality rates and the causative factors that lead to death [2,3]. One-year mortality rates vary among the studies, and they suggest that the survival rate is around 64-90% [4,5]. Both male and female patients after hip fractures have a higher mortality risk than the general population of similar age [6]. The many prognostic factors that the literature proposes may be related to death after hip fracture include age, male gender, comorbidities, American Society of Anesthesiologists (ASA) score, preinjury physical condition, cognitive status, type of fracture, type of surgery, time of delay to surgery, length of hospital stay, and albumin level [7,8]. Besides these standard factors, neutrophil-to-lymphocyte ratio (NLR) and platelet-to-lymphocyte ratio (PLR) were also newly defined parameters to predict mortality after hip fractures [9,10]. However, using all these standard and newly defined parameters to predict early mortality is still controversial, and no consensus has yet been reached. This study aimed to identify the standard and newly defined prognostic factors that could predict early mortality after treatment for hip fractures in elderly patients.

## Materials and methods

With the approval of the local ethics committee, the medical records for 481 patients aged ≥70 years who were admitted to our hospital between May 2017 and May 2019 due to low-energy femoral neck or intertrochanteric fractures were reviewed. All patients had been treated with hemiarthroplasty (cemented or uncemented) surgery or proximal femoral nailing surgery. All patients were followed up for at least one year after admission or until death. Exclusion criteria included having a pathologic fracture, a concomitant fracture, a periprosthetic fracture, high energy trauma, death before surgery, or having conservative treatment due to a high death risk with surgery. The patients who had data missing for variables other than laboratory data were excluded. The patients who were treated with total hip arthroplasty, proximal femoral plating, or osteosynthesis with dynamic hip screw were also excluded due to the insufficient number to provide reliable data. After applying these criteria, this observational study was performed using the records of 335 patients.

All data were collected from the hospital database. The outcome of interest was mortality, and survival data were obtained from the National Death Registry System in May 2020, which was the endpoint of the study. If the patient was already dead, the time of death was determined as the number of days from admission date until death. 

Determination of prognostic factors

The variables were selected according to their significance as indicated in previously published articles. Patients’ sociodemographic data (age, gender), presence of comorbidities, type of fracture (according to Arbeitsgemeinschaft für Osteosynthesefragen {AO} classification), implant type, American Society of Anesthesiologists (ASA) score, type of anesthesia, presence of delirium, time delay to surgery, length of hospital stay, length of stay in intensive care unit (ICU), units of erythrocyte transfusion, and laboratory data (preoperative hemoglobin, platelet count, neutrophil count, total leucocyte count, lymphocyte count, percentage of hemoglobin fall in the first postoperative day, preoperative albumin, blood urea nitrogen {BUN}, INR levels and newly defined neutrophil/lymphocyte ratio {NLR}, platelet/lymphocyte ratio {PLR} parameters) were all assessed in relation to mortality at 30 days, 90 days, or one year.

The neutrophil-to-lymphocyte ratio (NLR) is a newly defined variable and is calculated as the neutrophil count divided by the lymphocyte count. The platelet-to-lymphocyte ratio (PLR) is defined as the platelet count divided by the lymphocyte count.

Age groups were defined as aged 70-79, 80-89, and ≥ 90 years. The type of fracture was classified as either femoral neck or intertrochanteric. Intertrochanteric fractures were further classified as stable or unstable, according to AO classification. The intertrochanteric fractures that belonged to the classes 31A1.1, 31A1.2, 31A1.3, 31A2.1 were accepted as stable, and those in other classes were accepted as unstable. Three types of surgery (cemented hemiarthroplasty, uncemented hemiarthroplasty, and proximal femoral nailing) were included in this study. Regional and general anesthesia were compared as types of anesthesia. Time delay to surgery was defined as the number of days between admission and surgery, and patients were grouped into either within two days or after two days. Comorbidities were collected from patients' International Statistical Classification of Diseases (ICD)-10 codes or from preoperative consultations with anesthesiologists as recorded in the hospital database. Asthma, chronic obstructive pulmonary disease, ischemic heart disease, chronic anemia, hypertension, arrhythmia, Parkinson's disease, dementia, diabetes mellitus, thyroid pathology, peripheral vascular disease, and renal disease were the primary comorbidities. The comorbidity count was determined as having either 0-2 of them or ≥ 3.

Statistical analysis

Statistical analysis was carried out using Number Cruncher Statistical System (NCSS) 2007 software (Kaysville, UT: NCSS, LLC.). Descriptive statistical methods were used for means, standard deviation (SD), and percentages. Distribution of variables was determined by the Shapiro-Wilk normality test. All variables were tested by logistic regression analyses to investigate whether they affected 30-day mortality, 90-day mortality, or one-year mortality. Independent t-test was used for comparison of binary groups of variables showing normal distribution, and the Mann-Whitney U test was used if variables did not show a normal distribution. The chi-square test was used for the comparison of qualitative data. The statistically significant level was set at 0.05.

## Results

Among the 335 hip fracture patients, 199 (59.4%) were female and 136 (40.2%) were male. The mean age of the patients was 83.21 years (SD ± 11.9, range: 70-105 years) with 13.7% of them aged ≥ 90 years. Of the 335 patients, 36 (10.4%) were deceased within the first 30 days, 72 (21.5%) of patients within 90 days, and 113 (33.7%) of them were deceased by the end of one year.

Hip fractures were grouped into intertrochanteric fractures (254 patients, 75.8%) and neck fractures (81 patients, 24.2%). Intertrochanteric fractures were classified as either stable (123 patients, 48.6%) or unstable (130 patients, 51.4%). One intertrochanteric fracture was not classified due to the poor image quality. Two hundred and sixty (77.6%) patients were treated with hemiarthroplasty (179 intertrochanteric fracture patients, 81 neck fracture patients) and 75 (22.4%) patients were treated with proximal femoral nailing (all of them were intertrochanteric fracture). Of the hemiarthroplasty group, 40 (15.4%) patients were cemented and 220 (84.6%) patients were uncemented. 

Spinal anesthesia was administered to 284 (84.8%) patients and the other 51 (16.2%) patients were operated on with general anesthesia. Presence of delirium was observed in 51 (16.2%) patients. Intensive care was required by 184 (54.9%) patients. Two hundred and sixty-six (79.4%) patients were operated on within two days, the other 69 (20.6%) patients were operated on after a two-day period. The presence of ≥ 3 comorbidities was observed in 134 patients (40.1%). The baseline characteristics of the patients assessed are shown in Table 1. 

**Table 1:** Descriptive statistics of the patients SD: standard deviation; BUN: blood urea nitrogen; INR: international normalized ratio

Factors	N	Minimum	Maximum	Mean	SD
Age	335	70	105	83.21	6.10
Erythrocyte transfusions (unit)	335	0	16	1.76	1.71
Length of hospital stay (days)	335	1	178	12.03	15.08
Length of stay in ICU (days)	184	1	125	6.66	14.38
Delay to surgery (days)	335	0	7	1.58	1.36
Total leucocyte count	334	4.02	22.08	10.37	3.31
Preoperative hemoglobin (g/dl)	334	4.00	16.80	12.01	1.79
Postoperative hemoglobin (g/dl)	327	5.30	15.50	10.33	1.47
Postoperative hemoglobin fall (%)	327	-175.00	53.10	12.42	17.63
Platelet count(10^3^/µl)	334	71	806	239.56	86.67
Neutrophil count(10^3^/µl)	334	0.04	19.24	8.23	3.38
Lymphocyte count(10^3^/µl)	334	0.35	8.10	1.2837	0.81
Platelet/lymphocyte ratio (PLR)	333	45.19	948.24	237.34	143.30
Neutrophil/lymphocyte ratio (NLR)	334	0.03	60.00	8.8	6.59
INR	327	0.86	7.79	1.12	0.48299
Preoperative albumin (g/dl)	276	1.70	4.60	3.49	0.54556
BUN (mg/dl)	334	9.00	98.00	25.99	12.01

Thirty-day mortality

Univariate analysis showed that age, age ≥ 90 years, ASA score of 4, preoperative low albumin level, admission to ICU, and length of stay in ICU significantly increased the 30-day mortality rate (Table 2). Subsequently, logistic regression analysis was performed on the significant variables obtained from univariate analysis to determine 30-day mortality. Age ≥ 90 years (p-value: 0.013, odds ratio {OR}: 0.13) and ASA score of 4 (p-value: 0.019, OR: 0.22), independently, both had a significant impact on 30-day mortality (Table 3). All the other variables were not significantly associated with 30-day mortality.

**Table 2:** Univariate analysis of 30-day mortality, 90-day mortality, one-year mortality *Chi-square test. **Independent t-test. ***Mann-Whitney U test. ASA: American Society of Anesthesiologists; ICU: intensive care unit; NLR: neutrophil/lymphocyte ratio; BUN: blood urea nitrogen; INR: international normalized ratio

Factors		30-day mortality				p-Value	90-day mortality				p-Value	One-year mortality				p-Value
		Alive, n: 300		Dead, n: 35			Alive, n: 263		Dead, n: 72			Alive, n: 222		Dead, n: 113		
Implant type	Hemiarthroplasty	230	76.67%	30	85.71%	0.224*	196	74.52%	64	88.89%	0.01*	165	74.32%	95	84.07%	0.043*
	Proximal femoral nailing	70	23.33%	5	14.29%		67	25.48%	8	11.11%		57	25.68%	18	15.93%	
Implant type	Uncemented hemiarthroplasty	196	65.33%	24	68.57%	0.353*	166	63.12%	54	75.00%	0.035*	139	62.61%	81	71.68%	0.126*
	Cemented hemiarthroplasty	34	11.33%	6	17.14%		30	11.41%	10	13.89%		26	11.71%	14	12.39%	
	Proximal femoral nailing	70	23.33%	5	14.29%		67	25.48%	8	11.11%		57	25.68%	18	15.93%	
Cement	Absent	196	85.22%	24	80.00%	0.456*	166	84.69%	54	84.38%	0.951*	139	84.24%	81	85.26%	0.826*
	Present	34	14.78%	6	20.00%		30	15.31%	10	15.63%		26	15.76%	14	14.74%	
Age		82.72±5.9		87.49±6.24		0.0001	82.39±6.04		86.24±5.37		0.0001**	82.02±6.1		85.56±5.42		0.0001**
Age	70-79	82	27.33%	3	8.57%	0.0001*	78	29.66%	7	9.72%	0.0001*	71	31.98%	14	12.39%	0.0001*
	80-89	184	61.33%	20	57.14%		158	60.08%	46	63.89%		130	58.56%	74	65.49%	
	≥90	34	11.33%	12	34.29%		27	10.27%	19	26.39%		21	9.46%	25	22.12%	
Gender	Female	182	60.67%	17	48.57%	0.357*	162	61.60%	37	51.39%	0.240*	142	63.96%	57	50.44%	0.017*
	Male	118	39.33%	18	51.43%		101	38.40%	35	48.61%		80	36.04%	56	49.56%	
ASA score	1	3	1.01%	0	0.00%	0.001*	3	1.15%	0	0.00%	0.023*	3	1.36%	0	0.00%	0.0001*
	2	120	40.27%	11	31.43%		109	41.76%	22	30.56%		101	45.91%	30	26.55%	
	3	167	56.04%	18	51.43%		142	54.41%	43	59.72%		110	50.00%	75	66.37%	
	4	8	2.68%	6	17.14%		7	2.68%	7	9.72%		6	2.73%	8	7.08%	
Comorbidities	0-2 comorbidities	182	60.87%	18	51.43%	0.281*	163	62.21%	37	51.39%	0.097*	143	64.41%	57	50.89%	0.017*
	≥3 comorbidities	117	39.13%	17	48.57%		99	37.79%	35	48.61%		79	35.59%	55	49.11%	
Delirium	Absent	253	84.33%	31	88.57%	0.509*	227	86.31%	57	79.17%	0.135*	195	87.84%	89	78.76%	0.029*
	Present	47	15.67%	4	11.43%		36	13.69%	15	20.83%		27	12.16%	24	21.24%	
Type of anesthesia	Spinal	257	85.67%	27	77.14%	0.184*	229	87.07%	55	76.39%	0.025*	193	86.94%	91	80.53%	0.123*
	General	43	14.33%	8	22.86%		34	12.93%	17	23.61%		29	13.06%	22	19.47%	
Erythrocyte transfusion (unit)		1.74±1.70		1.91±1.84		0.823***	1.63±1.56		2.22±2.12		0.095***	1.58±1.29		2.11±2.29		0.172***
Length of hospital stay (days)		12.35±15.76		9.26±6.50		0.274***	11.17±14.99		15.15±15.15		0.084***	9.67±7.20		16.66±23.32		0.015***
Length of stay in ICU (days)		6.64±15.42		6.77±7.25		0.014***	5.01±14.02		10.65±14.61		0.001***	3.01±3.15		12.22±21.43		0.0001***
Admission to ICU	Admission (-)	146	48.67%	5	14.29%	0.0001*	133	50.57%	18	25.00%	0.0001*	111	50.00%	40	35.40%	0.011*
	Admission (+)	154	51.33%	30	85.71%		130	49.43%	54	75.00%		111	50.00%	73	64.60%	
Delay to surgery		1.6±1.36		1.43±1.42		0.361***	1.56±1.33		1.65±1.49		0.898***	1.55±1.29		1.65±1.51		0.921***
Delay to surgery	≤2 days	238	79.33%	28	80.00%	0.926*	210	79.85%	56	77.78%	0.701*	178	80.18%	88	77.88%	0.622*
	>2 days	62	20.67%	7	20.00%		53	20.15%	16	22.22%		44	19.82%	25	22.12%	
Total leucocyte count (10^3/µl)		10.3±3.19		10.98±4.25		0.25	10.26±3.25		10.79±3.56		0.224**	10.31±3.27		10.50±3.42		0.627**
Preoperative hemoglobin (g/dl)		11.99±1.78		12.24±1.92		0.448	12.05±1.74		11.9±1.99		0.524**	12.03±1.70		12.00±1.96		0.894**
Postoperative hemoglobin (g/dl)		10.29±1.45		10.71±1.66		0.122	10.31±1.48		10.44±1.48		0.502**	10.29±1.44		10.42±1.56		0.452**
Percentage of hemoglobin fall (%)		12.55±18.1		11.42±13.12		0.276***	12.9±18.03		10.67±16.09		0.274***	12.77±18.74		11.77±15.28		0.352***
Preoperative platelet count (10^3^/µl)		240.67±87.11		230.09±83.49		0.495**	240.06±85.08		237.75±92.85		0.842**	238.66±87.96		241.33±84.48		0.791**
Preoperative neutrophil count (10^3^/µl)		8.14±3.28		8.99±4.19		0.161**	8.09±3.34		8.76±3.52		0.139**	8.15±3.42		8.39±3.34		0.553**
Preoperative lymphocyte count (10^3^/µl)		1.30±0.84		1.14±0.50		0.282**	1.3±0.81		1.22±0.82		0.431**	1.29±0.72		1.28±0.98		0.952**
Platelet/lymphocyte ratio (PLR)		236.11±141.18		247.85±162.19		0.771***	232.58±133.72		254.93±174.16		0.562***	231.07±132.11		249.74±163.1		0.568**
Neutrophil/lymphocyte ratio (NLR)		8.70±6.62		9.78±6.40		0.244***	8.36±5.89		10.45±8.54		0.042***	8.52±6.10		9.38±7.46		0.303***
Preoperative INR		1.12±0.51		1.11±0.13		0.877**	1.10±0.33		1.21±0.83		0.077**	1.10±0.36		1.16±0.66		0.275**
Preoperative albumin (g/dl)		3.53±0.52		3.23±0.63		0.003**	3.54±0.51		3.37±0.62		0.023**	3.57±0.52		3.37±0.57		0.003**
Preoperative BUN (mg/dl)		25.63±11.98		29.17±11.99		0.099**	24.97±11.26		29.75±13.89		0.003**	24.8±10.57		28.34±14.19		0.011**
Type of fracture	Trochanteric	227	75.67%	27	77.14%	0.847*	199	75.67%	55	76.39%	0.899*	170	76.58%	84	74.34%	0.651*
	Neck	73	24.33%	8	22.86%		64	24.33%	17	23.61%		52	23.42%	29	25.66%	
AO classification	Stable	114	50.44%	9	33.33%	0.093*	100	50.51%	23	41.82%	0.254*	84	49.41%	39	46.99%	0.717*
	Unstable	112	49.56%	18	66.67%		98	49.49%	32	58.18%		86	50.59%	44	53.01%	

**Table 3:** Logistic regression analysis for 30-day mortality ASA: American Society of Anesthesiologists; ICU: intensive care unit; OR: odds ratio; CI: confidence interval

Factors		β-Value	p-Value		OR	95% CI for OR	
						Lower	Upper
Age (years)	80-89	0.74	0.117	0.038	0.48	0.19	1.20
	≥90	2.07	0.013		0.13	0.03	0.65
ASA score	ASA 2	1.44	0.998	0.140	-	-	-
	ASA 3	1.30	0.061		0.27	0.07	1.06
	ASA 4	1.52	0.019		0.22	0.06	0.78
Admission to ICU		1.08	0.063	0.063	0.34	0.11	1.06
Albumin (g/dl)		-0.55	0.148	0.148	0.58	0.27	1.22

Ninety-day mortality

In univariate analysis, age, age ≥ 90 years, ASA score of 4, and general anesthesia were found to be predictors of 90-day mortality (p-value: 0.0001, 0.0001, 0.023, and 0.025, respectively). Proximal femoral nailing technique was a significant predictor for survival (p-value: 0.035). Admission to ICU and length of stay in ICU were both found to be predictors of 90-day mortality (p-value: 0.0001 and 0.001, respectively). Preoperative low albumin, high BUN levels, and the newly defined high NLR as laboratory variables were all significant for 90-day mortality (p-value: 0.023, 0.003, and 0.042, respectively) (Table 2). 

Following logistic regression analysis, age (p-value: 0.002), age of 80-89 years (p-value: 0.032, OR: 0.43), age ≥ 90 years (p-value: 0.001, OR: 0.13), general anesthesia (p-value: 0.016, OR: 0.41), high NLR (p-value: 0.028, OR: 1.05), and high BUN levels (p-value: 0.049, OR: 1.02) were all significant predictors for 90-day mortality (Table 4).

**Table 4:** Logistic regression analysis for 90-day mortality ASA: American Society of Anesthesiologists; ICU: intensive care unit; NLR: neutrophil/lymphocyte ratio; BUN: blood urea nitrogen; OR: odds ratio; CI: confidence interval

Factors		β-Value	p-Value		OR	95% CI for OR	
						Lower	Upper
Hemiarthroplasty		0.76	0.093		2.15	0.88	5.24
Age (years)	80-89	0.85	0.032	0.002	0.43	0.20	0.93
	≥90 age	2.02	0.001		0.13	0.04	0.41
ASA score	ASA 2	1.21	0.998	0.467	0.00	0.00	-
	ASA 3	1.02	0.116		0.36	0.10	1.29
	ASA 4	0.92	0.136		0.40	0.12	1.34
General anesthesia		0.88	0.016		0.41	0.20	0.85
Admission to ICU		0.43	0.238		0.65	0.32	1.33
NLR		0.05	0.028		1.05	1.01	1.09
Albumin (g/dl)		-0.27	0.369		0.76	0.42	1.38
BUN (mg/dl)		0.02	0.049		1.02	1.00	1.05

One-year mortality

Age (p-value: 0.0001), age ≥ 90 years (p-value: 0.0001), male (p-value: 0.017), hemiarthroplasty surgery (p-value: 0.043), presence of delirium (p-value: 0.029), presence of ≥ 3 comorbidities (p-value: 0.017), ASA score of 4 (p-value: 0.0001), length of hospital stay (p-value: 0.015), admission to ICU (p-value: 0.011), length of stay in ICU (p-value: 0.0001), preoperative low albumin (p-value: 0.003), and high BUN levels (p-value: 0.011) all reached statistical significance for one-year mortality in the univariate analysis conducted (Table 2).

In the subsequent logistic regression analysis, predictor factors for one-year mortality were determined as age ≥ 90 years (p-value: 0.003), length of hospital stay (p-value: 0.003), and preoperative low albumin levels (p-value: 0.037) (Table 5).

**Table 5:** Logistic regression analysis for one-year mortality ASA: American Society of Anesthesiologists; ICU: intensive care unit; BUN: blood urea nitrogen; OR: odds ratio; CI: confidence interval

Factors		β-Value	p-Value		OR	95% CI for OR	
						Lower	Upper
Hemiarthroplasty		0.58	0.125		1.79	0.85	3.77
Age (years)	80-89	0.47	0.225	0.006	0.62	-	-
	>90	1.46	0.003		0.23	0.09	0.60
Gender	Male	0.42	0.134		0.66	0.38	1.14
ASA score	ASA 2	0.21	0.999	0.443	-	-	-
	ASA 3	0.87	0.170		0.42	0.12	1.45
	ASA 4	0.46	0.443		0.63	0.20	2.05
Comorbidities (>3)		0.44	0.135		0.64	0.36	1.15
Presence of delirium		0.21	0.557		0.81	0.40	1.64
Length of hospital stay (days)		0.04	0.003		1.04	1.01	1.06
Admission to ICU		0.14	0.665		1.14	0.62	2.10
Albumin (g/dl)		-0.52	0.037		0.60	0.37	0.97
BUN (mg/dl)		0.01	0.798		1.00	0.98	1.03

## Discussion

In this study, which aimed to determine the prognostic factors for mortality in hip fracture patients, being aged ≥ 90 years and having an ASA score of 4 were found to be associated with 30-day mortality; age, being aged 80-89 years, being aged ≥ 90 years, general anesthesia, high NLR and BUN levels were all associated with 90-day mortality, while being aged ≥ 90 years, length of hospital stay, and preoperative albumin levels were associated with one-year mortality. Being aged 90 years or older was the only significant prognostic factor that affected all early mortality timings. Being a nonagenarian seems to be one of the strongest indicators for mortality [11,12]. Nonagenarians have more comorbidities than the younger elderly, and their functional levels and mobility are far more limited. The population of the world is aging, and the number of people who will reach their 90s is increasing. Therefore, fragility fractures of the hip will inevitably increase in the coming years. Reducing comorbidities, presenting a better public health service, and a routine follow-up for osteoporosis in the elderly will provide the opportunity to lower both early and total mortality rates. 

NLR is an inflammatory biomarker and has been proposed for use in some fields of medicine, such as gastrointestinal disease, oncology, and cardiology [13-15]. NLR was first used in hip fracture patients by Forget et al. [16]. They stated that admission NLR was not useful as a predictor for mortality and proposed NLR on the fifth postoperative day. In contrast, Fisher et al. showed that admission NLR was a significant independent risk factor in geriatric hip fractures for postoperative myocardial injury, high inflammatory response, and in-hospital death [9]. PLR is another proposed inflammatory biomarker, similar to NLR, and it has been studied in relation to adverse cardiac outcomes and oncology [17,18]. PLR was first investigated as a prognostic risk factor in hip fractures by Emektar et al. [10]. Although they initially stated that high PLR at admission was found to be predictive of one-year mortality, they concluded that it was clinically insignificant. In this study, we found that NLR was an independent prognostic risk factor, in both univariate and logistic regression analysis, for 90-day mortality, but it did not emerge as a significant prognostic factor for one-year mortality. PLR evaluation was consistent with the literature and was not found to be significant as a prognostic factor [10]. This study showed that the male gender had a significantly higher mortality rate in univariate analysis of 90-day mortality, but when logistic regression analysis was applied, it was not found to be as significant as previous studies had concluded [1]. In comparison, Kannegaard et al. and Endo et al. showed that male patients had an excess mortality rate compared to women [19,20]. In the present study, the patients were evaluated preoperatively with an ASA score, and an ASA score of 4 was associated with 30-day mortality in both the univariate and logistic regression analysis. Although an ASA score of 4 was also associated with 90-day mortality and one-year mortality, it did not remain significant after logistic regression analyses. In some studies, ASA score has been shown to be a prognostic factor for early mortality [21]. Other studies have denied that there is a correlation between mortality and ASA scores for patients over 90 years [22]. It is a well-accepted fact that increased comorbidities bring higher mortality rates. Although the presence of ≥ 3 comorbidities was significant for one-year mortality in the univariate analysis, this parameter did not remain significant. Due to the retrospective nature of this study, the comorbidities of the patients could not be perfectly determined.

The effect of anesthetic technique on early mortality has been researched in numerous studies, and the majority of them demonstrate that general anesthesia is a negative prognostic factor [23]. In contrast, a Cochrane review in 2016 showed that type of anesthesia had no effect on one-year mortality [24]. In another review, Van Waesberghe et al. investigated whether type of anesthesia affected 30-day mortality, and no significant difference was found [25]. In our study, general anesthesia was associated with higher 90-day mortality rate after applying logistic regression analysis. We found no relation with 30-day or one-year mortality, in agreement with the literature.

The mortality rate did not differ with the type of fracture (neck vs intertrochanteric, stable intertrochanteric vs unstable intertrochanteric). Although hemiarthroplasty surgery was associated with 90-day and one-year mortality, after logistic regression analysis, it was not significant. Neither cemented nor uncemented hemiarthroplasty was superior to the other. The literature is controversial regarding the type of fracture and the type of implant [7].

Hip fracture patients are at risk for confusion and delirium due to trauma. Dolan et al. found admission delirium to be an important factor for functionality and poor outcomes [26]. They stated that it did not remain as a significant predictor for mortality after adjustment for confounding variables. However, opposite results have also been indicated [27]. In the present study, delirium was associated with 90-day mortality in the univariate analysis but was not associated with mortality after logistic regression analysis.

A longer in-hospital stay was significantly associated with one-year mortality. We determined length of hospital stay as a significant prognostic factor for one-year mortality. Nikkel et al. stated that increased length of stay resulted in an increased mortality rate in a US cohort [28]. The findings of our study and those of Nikkel et al. contrast sharply with a Swedish study by Nordström et al., in which shortened hospital stay was found to be a risk factor for mortality [29]. 

Preoperative serum albumin level, which identifies the nutritional status of patients, is one of the most researched variables for early mortality. Studies show that hypoalbuminemia (≤ 3.5 g/dl) is associated with one-year mortality [30]. Our data confirmed this, and it was determined as a risk factor for one-year mortality in our study. Chronic renal failure is thought to be a prognostic risk factor for early mortality. Although high BUN levels, which are an indicator of chronic renal failure, were associated with 90-day mortality in this study, it should not be considered a valuable or reliable prognostic factor due to its susceptibility to diet and muscle loss. We found no significant relation between early mortality and the variables of delay before surgery, the number of erythrocyte transfusions, preoperative hemoglobin, percentage of hemoglobin fall, platelet count, lymphocyte count, total leucocyte count, neutrophil count, or preoperative INR. 

This study has a few limitations. It was not possible to obtain some of the laboratory data or the preoperative mobility status of the patients due to the retrospective nature of the study. The importance of preoperative and postoperative mobilization levels of the patients is known for postoperative mortality and morbidity. Because of the retrospective course of our study, we could not exactly evaluate the mobilization level. Other treatment modalities, such as total hip arthroplasty, osteosynthesis with proximal femoral plating, or dynamic hip screw were also excluded due to the lesser number of patients operated with these treatment methods. Numerous factors for mortality have been proposed, but we were only able to investigate the most important of these factors. This is another limitation of the this study.

## Conclusions

In conclusion, a detailed assessment of risk factors is crucial in order to reduce the mortality rate after hip fractures. Our data suggest that being aged ≥ 90 years, having an ASA score of 4, general anesthesia, high NLR ratio, high BUN levels, longer length of hospital stay, and preoperative low albumin levels are independent risk factors for early mortality. We suggest that NLR, which is a new and cheap prognostic biomarker, should continue to be researched extensively in prospective studies with larger sample sizes.
